# Quality of antenatal care in rural southern Tanzania: a reality check

**DOI:** 10.1186/1756-0500-3-209

**Published:** 2010-07-27

**Authors:** Malabika Sarker, Gerhard Schmid, Elin Larsson, Sylvia Kirenga, Manuela De Allegri, Florian Neuhann, Theodora Mbunda, Isaack Lekule, Olaf Müller

**Affiliations:** 1 Institute of Public Health, University of Heidelberg, Heidelberg, Germany; 2 Swiss Tropical Institute, University of Basel, Basel, Switzerland; 3 Division of Global Health, Department of Public Health Sciences, Karolinska Institute, Stockholm, Sweden; 4 Ifakara Health Institute, Dar es Salaam, Tanzania

## Abstract

**Background:**

Counselling on the danger signs of unpredictable obstetric complications and the appropriate management of such complications are crucial in reducing maternal mortality. The objectives of this study were to identify gaps in the provision of ANC services and knowledge of danger signs as well as the quality of care women receive in case of complications.

**Findings:**

The study took place in the Rufiji District of Tanzania in 2008 and was conducted in seven health facilities. The study used (1) observations from 63 antenatal care (ANC) sessions evaluated with an ANC checklist, (2) self-assessments of 11 Health workers, (3) interviews with 28 pregnant women and (4) follow-up of 12 women hospitalized for pregnancy-related conditions.

Blood pressure measurements and abdominal examinations were common during ANC visits while urine testing for albumin or sugar or haemoglobin levels was rare which was often explained as due to a lack of supplies. The reasons for measuring blood pressure or abdominal examinations were usually not explained to the women. Only 15/28 (54%) women were able to mention at least one obstetric danger sign requiring medical attention. The outcomes of ten complicated cases were five stillbirths and three maternal complications. There was a considerable delay in first contact with a health professional or the start of timely interventions including checking vital signs, using a partograph, and detailed record keeping.

**Conclusion:**

Linking danger signs to clinical and laboratory examination results during ANC with the appropriate follow up and avoiding delays in emergency obstetric care are crucial to the delivery of coordinated, effective care interventions.

## Background

Complications like haemorrhage, sepsis and hypertensive disorders account for most of the maternal deaths in developing countries [1]. Key interventions to reduce maternal deaths include the promotion of antenatal care (ANC), presence of skilled assistants during childbirth and provision of emergency obstetric care [2]. The cornerstones of effective ANC and timely referrals during complications are the identification of pregnancy complications and women's awareness of the danger signs [2-7]. Although certain obstetric emergencies cannot be predicted through antenatal screening, women can be educated to recognize and act on symptoms that potentially lead to serious conditions [8]. Therefore, the continuity of care starting from raising awareness about danger signs at first level facilities to recognition of such signs at household level to access and receipt of the appropriate care for such complications at referral health facilities is crucial in reducing maternal deaths.

The maternal mortality ratio (MMR) among Tanzanian women is still high (578/100,000) [9]. The high MMR in Tanzania, despite good antenatal and delivery care coverage, highlights the issue that the use of maternal health services alone is not sufficient to reduce maternal mortality. Studies report that very few women in rural Tanzania can identify three or more obstetric danger signs [4,10]. Also, women are reluctant to seek care at health facilities due to prior negative experiences with health workers [11-13]. In addition, the quality of care in health facilities has been reported to be very poor [14-16].

The health outcome of women admitted to the health facility after recognition of the danger signs is ultimately dependent upon the care received. Improving the quality of care requires continuity of the care and it is important to know whether women are informed about danger signs and understand the importance of it, and what happens when a woman presents herself in the health facility with such signs. Thus, the aim of this paper is to report the results of a study that examined gaps in the provision of ANC services, knowledge of danger signs and appropriate obstetric care in health facilities in rural Tanzania.

## Methods

### Settings

This study was conducted in the Rufiji District, situated south of Dar es Salaam, which is home to several different ethnic groups [17]. Maternal health care in Rufiji is provided at three hospitals (faith-based, private, and government), five health centres and 48 dispensaries. This study is part of a larger research project on the Effects of Antiretroviral for HIV on African Health Systems, Maternal and Child health (ARVMAC, http://www.arvmac.eu).

### Data collection

This cross-sectional and health facility-based study was conducted from January to June 2008. To represent all levels of care, seven health facilities were purposely selected for inclusion in the study: two hospitals (government and mission hospital), two health centres and three dispensaries. The study applied both quantitative and qualitative data collection that included four components: (1) observations from 63 antenatal care (ANC) sessions evaluated with an ANC checklist, (2) self-assessments of 11 Health workers, (3) interviews with 28 pregnant women and (4) follow-up of 12 women hospitalized for pregnancy-related conditions.

The non-participatory structured observations were part of larger quantitative study that systematically assessed the quality of ANC services in three African countries. The ANC sessions were observed by a research assistant (RA) with a medical background.

The self assessment questionnaires listing ANC procedures were given to the health workers after the end of the ANC session. Health providers reported their performance by marking the checklist. Eleven health workers responsible for ANC (eight public health nurses, one midwife, one clinical officer, and one nursing officer) from all seven health facilities filled out a total of 35 questionnaires.

For the semi structured interviews, the RA, as the initial contact person, selected the pregnant women on the basis of number of ANC visits and pregnancies. After attending ANC, the selected women were approached by another interviewer and informed consent sought for the subsequent interviews. In total, 15 primi and 13 multi gravida women were interviewed, among them 13 who had come for their first visit.

The self assessment questionnaires completed by the health workers and semi-structured interviews of the pregnant women were linked to the structured observations. The information collected from observed ANC sessions were triangulated with the responses of the pregnant women that participated in the semi-structured interviews (28) and the self assessment questionnaires (35) of the health workers.

The follow up component of the study of 12 pre selected cases took place in the government and the mission hospitals. Two cases from each of the following obstetric categories were selected: pre-eclamptic toxaemia, prenatal eclampsia, postnatal eclampsia, haemorrhage, obstructed labour, and normal labour pain. The eligible women chosen for the study were admitted to the hospitals within a time period of four weeks.

The original English tools developed by local and northern investigators were translated into Kiswahili. To assess the accuracy of the translated questionnaire, the Kiswahili version was back-translated into English. All tools were pilot tested before use in the main study.

### Data Analysis

Quantitative data (ANC observations, self-assessment questionnaires) were analyzed using Excel and SAS 9.0 (SAS Institute Inc., Cary, NC, USA). Descriptive analyses were conducted to identify the proportion of tasks completed by the health workers. All individual interviews were transcribed and translated into English. Qualitative data analysis of the information from the semi-structured interviews was completed using NVivo 8.0 software (QSR International Pty Ltd 2007). The transcribed text was read multiple times by the first and third author. Emergent themes were identified by independent analysis. Similarities and differences between sub-groups were examined and compared.

The Ethical Committees of the Medical School, University of Heidelberg, Germany and Ifakara Health Institute (IHI), Dar es Salam, Tanzania, approved the study. In the Rufiji District, further approval was acquired from the District Medical Officer (DMO). Individual verbal informed consent was obtained from all study participants before conducting the interviews.

## Results

### Structured observation

The results from ANC observations show that health workers performed the majority of clinical examinations on the checklist including blood pressure checks (94%) and uterine height measurements (100%). However, urine tests for albumin (8%) and glucose (9%) were largely omitted. Only 61% women received information regarding danger signs (Table 1).

**Table 1:** Comparison of health worker (HW) responses and antenatal care (ANC) observations according to national guidelines (n = 35)

*Services provided *	*Service provision observed (%)*	*Self report* *by HW (%)*	*National guideline* *standard (4 visits)*
***Physical examination***			
Weight	24 (68)	31 (88)	Check every visit
Blood pressure	33 (94)	35 (100)	Check every visit
Anaemia	21 (60)	33 (94)	Check every visit
Oedema	12 (34 )	29 (83)	Check every visit
Uterine height	35 (100)	35 (100)	Check every visit
Vaginal/Vulva inspection **Δ**	10 (55)	20 (111 )	Check 1^st ^and 4^th ^visit
Foetal heart sound	31 (88)	35 (100)	Check >16w GA

***Laboratory examination***			
Haemoglobin	9 (26)	35 (100)	Check every visit
Urine for albumin	3 (08)	22 (63)	Check every visit
Urine for glucose	2 (09)	15 (43)	Check every visit
Syphilis	35 (100)	35 (100)	Check once
HIV	33 (94)	33 (94)	Check once
Blood group	12 (34)	12 (34)	Check once

***IEC***			
Danger signs	22 (61)	35 (100)	Inform every visit
Immunization	34 (97)	33 (94)	Inform (at least) once
Nutrition	33 (94)	31 (88 )	Inform every visit
Sexually transmitted infection information	11 (31)	32 (91 )	Inform every visit
Harmful substances and habits	20 (57 )	26 (74)	Inform every visit
PMTCT	31 (88)	31 (88)	Inform (at least) once

***Drugs administered ***			
Iron/Folate	16 (46 )	23 (66 )	Administer every visit
Antimalarial drug	24 (68)	31 (88)	Administer three times

***Reminded*** Client - next ANC visit	35 (100)	33 (94)	Inform every visit

### Self assessments

Health workers reported completing most of the tasks outlined in the ANC guidelines. Nonetheless, key discrepancies were detected between the observed ANC sessions and the provider's self assessments for the majority of the physical and laboratory examinations. Disparities were also noted regarding the provision of information, education and communication (IEC) to women during the visits (Table 1). Health workers did not inform five women of the main danger signs during their observed visit, stating that they had already done so in a previous visit, even though it is recommended that women be informed at every visit.

The irregular supply of drugs or reagents was reportedly one of the most important reasons for not conducting certain tests or distributing drugs. The perception that the service was not important was the reason frequently given for not checking oedema or measuring weight. Antimalarial prophylaxis, iron, and folic acid tablets were not routinely offered to every pregnant woman.

### Interviews with pregnant women

Interviews with the pregnant women revolved around three themes: a) Clinical and laboratory service provision, b) Quality of the information received and c) Health facility or home delivery decisions.

### Theme 1: Clinical and laboratory service provision

The services offered to women varied across health facilities. Most women reported that their blood pressure was checked and their uterine height was measured during their visit. Nonetheless, even though 24/28 (86%) had their blood pressure checked, 16/24 (67%) could not explain why the procedure was actually done.

*'The nurse wrapped a cuff on my arm and listened through another instrument. I don't know why and I didn't ask.' ***(Health centre, 28 yrs, fourth pregnancy, second visit)**.

Only 14/28 women (50%) were clinically checked for anaemia and 10/28 (36%) for oedema. Urine was tested for 2/28 (7%) of the women during their current visit. Women were not informed about the reasons for blood or urine tests and were reluctant or afraid to ask for any explanations.

### Theme 2: Quality of information received

Only 15/28 (54%) of the women were able to cite at least one of the danger signs (bleeding, or headache or discharge) requiring medical attention. Although all women received dietary information only 11/28 (40%) received information on birth planning.

*'She told me about delivery preparation. I should organize money for transport, food, buying gloves and blades. Also she told me about nutrition, danger symptoms in pregnancy'*. **(Dispensary, 17 years, first pregnancy, third visit)**.

### Theme 3: Health facility or home delivery decisions

The majority of the women (23/28, 82%) reported a willingness to deliver in a health facility. The most frequently mentioned reason was the necessity of being close to surgical or other forms of support.

*'I would like to deliver in a hospital because services to help during difficult delivery like operation and blood transfusion are available.' ***(Government hospital, 25 yrs, third pregnancy, third visit)**.

Reasons for preferences for home delivery were the costs associated with delivering in a health facility and the influence of traditional birth attendants (TBAs).

'I would like to deliver at home because there are many TBAs. Also it is far from my home to hospital and I pay TBA less, only 3,000 ( 2 USD Dollar)*^1 ^*and at Ikwiriri mission I have to pay Tsh 18, 000 (12 USD Dollar) for normal delivery and TSh 20, 000 (13 USD Dollar) for an operation (One USD dollar = 1500 Tsh at time study was conducted). **(Government hospital, 16 yrs, first pregnancy, sixth visit)**.

### Case studies

Out of the 12 follow up cases (ten with complications and two with labour pain), seven women had been referred from a lower level health facility. Eleven women had used ANC services more than twice and all except one carried an ANC card. Considerable delays were observed in starting treatment for eclampsia and haemorrhage, two of the most common life threatening conditions. Partographs required to monitor progress in delivery were not filled up in any of the hospitals. Other gaps noted were delays in admission, consultation and intervention. Additionally, deficiencies in immediate basic care such as checking vital signs and abdominal examinations as well as record keeping, monitoring and follow up were also observed. The lack of documentation and absence of notes on activities were more common in the government hospital compared to the mission hospital (Table 2).

**Table 2:** Characteristics of selected cases of pregnant women followed up

No	Health facility Diagnosis	Distance to HF (km)	Transport/ Travel time (hours)	Gaps identified during care
1	Gov. Hospital Prenatal eclampsia & Antepartum haemorrhage	2	On foot 0.30 h	→ 11 hours delay in care → 14 hours after admission: CS → No: follow up, document, partograph
2	Gov. Hospital Postnatal eclampsia	1	On foot 0.15 h	→ 6 hours delay in care → No documentation → Fell down on the floor due to convulsion
3	Gov. Hospital Pre eclamptic toxaemia	40	Bicycle 9.0 h	→ Immediate care, delivery after 4 hours → No: BP check up → No partograph.
4	Missionary Hospital Pre eclamptic toxaemia	< 1	On foot 0.10 h	→ Diagnosed-high BP in dispensary → No urine test for albumin → Twice sent home, 10 days delay → Referred to missionary hospital → Immediate care, Caesarian section
5	Gov. Hospital Obstructed labour	< 1	On foot 0.10 h	→ 7 hours delay - first visit by doctor → 10 hours after admission CS done → No: document, follow up, partograph, → Baby had umbilical cord sepsis
6*	Gov. Hospital Obstructed labour	1	On foot 0.15 h	→ 2 hrs delay, first visit nurse → 2 hrs 45 m after visit of doctor CS done → No: follow up, document.
7	Gov. Hospital Antepartum haemorrhage	12	Bi cycle 3.30 h	→ Advised for ultrasonography (US), Sonographer absent, delay ~24 h, → Discharged, returned after 10 days → US done, again discharged by clinician. → Admitted after 3 days with shock, → CS done, No: follow up, document.
8	Mission Hospital Antepartum haemorrhage	> 40	Boat, Car > 10 h	→ Immediate care → Ultrasonography, Caesarean section → Good post natal care.
9	Health Centre Mission Hospital Post partum haemorrhage	50	Bicycle, on foot > 13 h	→ Immediate care → Retained placenta was already removed in the health centre
10	Miss. Hospital Post partum haemorrhage	> 80	Car > 5 h	→ Immediate care
11	Mission Hospital Labour pain	3	On foot 0.45 h	→ Immediate care → No partograph.
12	Mission Hospital Labour pain	2	On foot 0.30 h	→ Immediate care → No partograph

Among the ten women admitted with complications, five had stillbirths. Three women who lost their babies also suffered from urine incontinence, puerperal sepsis and perineal tears (Table 3).

**Table 3:** Diagnosis, intervention and outcome of cases

**No**.	**Diagnosis by health professional **	**Intervention**	**Outcome** **mother**	**Outcome baby**	**Complication**
**1**	Prenatal eclampsia + Antepartum haemorrhage	Caesarean section	Alive	Still birth	Temporary renal failure + urinal incontinence
**2**	Postnatal eclampsia	Vaginal delivery	Alive	Macerated still birth	Perineal tears + puerperal sepsis
**3**	Pre eclamptic toxaemia	Vaginal delivery	Alive	Stillbirth	Perineal tears
**4**	Pre eclamptic toxaemia	Caesarean section	Alive	Alive	Nil
**5**	Obstructed labour	Caesarean section	Alive	Alive	Umbilical sepsis
**6**	Obstructed labour + foetal distress)	Caesarean section	Alive	Alive	Nil
**7**	Antepartum haemorrhage	Caesarean section Blood transfusion	Alive	Stillbirth	Nil
**8**	Antepartum haemorrhage	Caesarean section	Alive	Still birth	Nil
**9**	Post partum haemorrhage due retained placenta	Bi-manual removal of placenta	Alive	Alive	Nil
**10**	Post partum haemorrhage due to atonic uterus	Oxytocin, Blood transfusion	Alive	Alive	Nil
**11**	Normal delivery	Vaginal delivery	Alive	Alive	Nil
**12**	Normal delivery + foetal distress	Vaginal delivery	Alive	Alive	Nil

## Discussion

This paper highlights several gaps in the care and information provided regarding danger signs. The results showed that during ANC visits, health providers omitted certain practices stipulated in national ANC guidelines. Blood pressure measurements and abdominal examinations were common while urine testing for albumin or sugar and checks of haemoglobin levels were rare, although these are routine tests required for all pregnant women, according to WHO and Tanzanian Government guidelines [16,18]. The main reason given for not performing these tests was a shortage of supplies, as previously observed in a study in Kenya [19].

The findings were further corroborated by the statements made by the women. The reasons for conducting the clinical examinations were not explained to the women. Furthermore, as found in studies in other African countries, the majority of the women were only able to cite two danger signs requiring medical attention: severe headache and vaginal bleeding [4,10]. Not all women were informed about birth planning with skilled attendants as a means to increase delivery success, which has also been reported from another study in Tanzania [20].

These results confirm that despite high ANC attendance, women are not fully benefiting from IEC, the primary purpose of ANC [21,22]. Inadequate understanding of the purpose of physical checkups and lack of knowledge of danger signs may lead to delays in seeking appropriate medical care or even failure to arrive at health facilities when complications arise. Women equipped with better knowledge can motivate their family members to play a major role in the healthcare-seeking behaviour of pregnant women, particularly in emergency situations [23]. Consistent with the findings from another study in Tanzania, we observed that women were committed to investing in ANC and valued the importance of delivering in a health facility but were unable due to financial constraints [24].

The poor quality of emergency obstetric care observed during follow up of the case studies, especially in government health facilities, is a major concern. Inadequacies in information recording can hinder the monitoring of a patient's progress. Failure to use partographs was common in both public and private facilities. This was also observed in a Dar es Salaam hospital, where 50% of the cases did not receive a partogram. Furthermore, evidence existed of substandard vital sign recording in those partograms that were performed [25].

In this study, five out of the ten women admitted with complications delivered a stillbirth. Three of these five women suffered from some form of morbidity, consistent with reports from other parts of Tanzania [15]. These findings also suggested that to reduce maternal morbidity and neonatal deaths timeliness and adequate care for women who seek emergency care needed to be ensured.

Certain limitations in the current study must be acknowledged. First, the non-participatory observations by the RA may have influenced the performance of health workers in a positive direction. Second, the health worker self assessments were reflective of individual workloads and willingness to participate in the study, which could have led to bias. Finally, the nursing background of the observer in the hospitals may have directly or indirectly influenced patient outcomes.

In conclusion, during ANC visits health workers need to link patient information to clinical and laboratory outcomes so that women can understand, later recognize and act on symptoms. This may accelerate the timeliness of referrals, thus avoiding the consequences associated with potentially serious conditions. In addition, the regular supply of materials and provision of supervision and refresher training for health workers should significantly improve the quality of maternal health services. There is also a need to improve the quality of ritually performed diagnostic procedures during ANC sessions, as seen in comparable settings.

## Competing interests

The authors declare that they have no competing interests.

## Authors' contributions

SM, SG, and DM conceptualized the study and developed the tools. SG and KS performed the literature review. SM and LE analyzed qualitative data, SG and NF analyzed quantitative data. TM and IL coordinated, collected, and entered the data. SM and MO drafted and finalized the manuscript. All authors read and approved the final manuscript.
